# SARS-CoV-2 Short-Time Infection Produces Relevant Cytopathic Effects in Vero E6 Cell Line

**DOI:** 10.3390/ijerph18179020

**Published:** 2021-08-26

**Authors:** Luisa Zupin, Francesco Fontana, Rossella Gratton, Margherita Milani, Libera Clemente, Lorella Pascolo, Maurizio Ruscio, Sergio Crovella

**Affiliations:** 1 Institute for Maternal and Child Health IRCCS Burlo Garofolo, 34137 Trieste, Italy; rossella.gratton@burlo.trieste.it (R.G.); lorella.pascolo@burlo.trieste.it (L.P.); 2 Division of Laboratory Medicine, University Hospital Giuliano Isontina (ASUGI), 34129 Trieste, Italy; francesco.fontana@asugi.sanita.fvg.it (F.F.); libera.clemente@asugi.sanita.fvg.it (L.C.); maurizio.ruscio@asugi.sanita.fvg.it (M.R.); 3 Department of Medicine, Surgery and Health Sciences, University of Trieste, 34149 Trieste, Italy; margherita.milani@studenti.units.it; 4 Department of Biological and Environmental Sciences, College of Arts and Sciences, University of Qatar, Doha 2713, Qatar; sgrovella@qu.edu.qa

**Keywords:** SARS-CoV-2, infection, Vero E6 cells

## Abstract

Severe acute respiratory syndrome coronavirus type 2 (SARS-CoV-2) is mainly transmitted through respiratory droplets from positive subjects to susceptible hosts or by direct contact with an infected individual. Our study focuses on the in vitro minimal time of viral absorption as well as the minimal quantity of virus able to establish a persistent infection in Vero E6 cells. We observed that 1 min of in vitro virus exposure is sufficient to generate a cytopathic effect in cells after 7 days of infection, even at a multiplicity of infection (MOI) value of 0.01. Being aware that our findings have been obtained using an in vitro cellular model, we demonstrated that short-time exposures and low viral concentrations are able to cause infection, thus opening questions about the risk of SARS-CoV-2 transmissibility even following short contact times.

## 1. Introduction

The primary mode of transmission of severe acute respiratory syndrome coronavirus type 2 (SARS-CoV-2) is through exposure to infective respiratory droplets and/or by direct contact with an infected person [1]. SARS-CoV-2 enters the human organism through the naso-oropharyngeal region, from where the virus can reach the lower airways, hence leading to the onset of the characteristic respiratory symptoms. Indeed, the coronavirus disease 2019 (COVID-19) causes cough, difficulty breathing, sore throat, and in more severe cases, pneumonia and acute respiratory distress syndrome (ARDS) [2].

SARS-CoV-2 presents a specific host cellular tropism. The entry of SARS-CoV-2 into susceptible cells is mediated by the interaction of the viral spike S protein with the angiotensin-converting enzyme 2 (ACE2) cellular receptor; then, S protein is cleaved by host proteases, allowing the virus–host membrane fusion [3]. Indeed, this virus is able to primarily infect ACE-2-expressing cells in the airways, inducing a direct cytopathic with consequent respiratory symptoms. It has been shown that SARS-CoV-2 presents an increasing gradient of infection from distal to proximal respiratory primary cell lines. Moreover, from autoptic findings, it has been observed that ciliated cells in the airways and pneumocytes type 2 of the alveoli are the cells more susceptible to infection. Moreover, cell death has also been reported as caused by the exacerbated immune response activated by the infection [4].

A meta-analysis estimated that both the basic reproduction number R0 and the effective reproductive number of SARS-CoV-2 are ~3; therefore, according to this meta-analysis, one person can infect three other individuals. However, these numbers are decreasing worldwide thanks to the containment measures undertaken by most governments [5]. Nevertheless, second and third waves of infections have occurred or are currently ongoing.

Crowded indoor environments represent remarkably high-risk situations, potentially favoring viral spread [6]. The World Health Organization (WHO) international guidelines of contact tracing designated that 15 min of contact with an infected person at less than 1 m is the critical time and space to SARS-CoV-2 exposure able to generate an infection. In addition, other conditions in scenarios involving close contacts have also been defined and include the direct physical contact with a COVID-19 positive individual, a COVID-19 patient-caregiver not wearing the proper protection, the sharing of a house, room, and/or meal with positive persons [7]. These settings could easily occur in different situations, for instance in the school classroom and public transports. Moreover, a critical setting happens in hospitals, where the prevention of SARS-CoV-2 transmission is a dramatical challenge both for health operators (e.g., nurses, doctors), hospitalized patients, and outpatients [8].

SARS-CoV-2 was generally isolated in vitro through the infection of permissive cell lines. Different cells can be employed, with the Vero cells (from the kidney of *Cercopithecus aethiops*) being the most employed for their high susceptibility to the replication of a wide range of viruses, including SARS-CoV-2. Few other human cell lines are tolerant to SARS-CoV-2 reproduction, including Calu-3 (lung adenocarcinoma), Caco-2 (colorectal adenocarcinoma), and Huh7 (hepatocellular carcinoma); nevertheless, in these human cells, the cytopathic effect was not evident as that observed in monkey cells [9,10]. Moreover, primary cells from human nasal epithelia, bronchi and large airway epithelia, bronchiolar and small airway epithelia, type II and type I pneumocytes are also valuable in vitro models for SARS-CoV-2 research [4]. Finally, more sophisticated approaches are represented by airway epithelial cells cultured in air-liquid interface and lung organoids [11].

The time of inoculation is generally set at 1–2 h [10]; however, to date, a study focusing on shorter time of virus inoculation, experimentally mimicking the 15 min of contact designated by WHO, is lacking [7].

Trying to fill this gap of knowledge, in this work, we aimed at determining the minimal time of absorption and the minimal quantity of virus able to produce a persistent infection in an in vitro cellular model.

## 2. Materials and Methods

Epithelial cell line Vero E6 cells from *Cercopithecus aethiops* normal kidney (ATCC CRL-1586) were employed in the experimental settings. Cells were maintained in MEM + 10% fetal bovine serum, 2 mM glutamine, and 100 U/mL penicillin/streptomycin (Euroclone, Pero, Italy). The day prior to the experiments, cells were seeded at a density of 5 × 10^4^ cells/well in 24 multiwell plates. SARS-CoV-2, previously isolated in the BLS3 facility of the San Polo Hospital (ASUGI, Monfalcone, GO, Italy) was employed in the experiments.

To determine the minimal time of viral adsorption and the minimal viral quantity able to establish a persistent infection, the virus was diluted in MEM + 2% fetal bovine serum, 2 mM glutamine, and 100 U/mL penicillin/streptomycin (Euroclone) at a multiplicity of infection (MOI) of 1, 0.1, 0.01, 0.001, and 0.0001 and then transferred to Vero E6 cells for 1, 2, 3, 5, 15, 30, or 60 min at 37 °C 5% CO_2_. At the end of the incubation, the virus was removed, the cells were washed in phosphate-buffered saline (PBS), and 1 mL of new culture medium was added to the wells (MEM + 2% fetal bovine serum, 2 mM glutamine, and 100 U/mL penicillin/streptomycin, Euroclone).

Fifteen microliters of supernatants were harvested at 7 days post-infection and then were subjected to thermolysis (98 °C for 3′ and 4 °C for 5′) with 45 microliters of distilled water for RNA extraction.

The viral load was quantified using real-time PCR with the Luna^®^ Universal Probe One-Step RT-qPCR Kit (New England Biolabs, Ipswich, MA, USA), with CDC primers and probe (Eurofins, Luxembourg) for the viral gene *N* (nucleocapsid, 500 nM forward primer GGG AGC CTT GAA TAC ACC AAA A, 500 nM reverse primer TGT AGC ACG ATT GCA GCA TTG, 125 nM probe FAM-AYC ACA TTG GCA CCC GCA ATC CTG-BHQ1) on the 7500 Fast Real-Time PCR system (Thermo Fisher Scientific, Waltham, MA, USA, protocol: 50 °C for 10′, 95 °C for 1′, and then 40 cycles at 95° for 10″, 60° for 30″). The previously quantified nCoV-CDC-Control Plasmid (Eurofins) was employed to generate the standard curve.

The cytopathic effect was monitored with the EVOS XL Core Cell Imaging System (Thermo Fisher Scientific, Waltham, MA, USA). At the end of the 7 days post-infection, supernatants were removed, and cells were fixed in 4% paraformaldehyde, diluted in phosphate-buffered saline (PBS) for 20 min, and then stained with 10% crystal violet diluted in PBS for 30 min. After the staining procedure, the wells were air-dried.

Finally, the presence of SARS-CoV-2 was assessed by immunofluorescence assay through the recognition of the N protein. Briefly, cells were grown on glass coverslips. At the end of the infection setting (7th days post virus exposition), wells were washed in PBS, and cells were fixed in 4% paraformaldehyde for 20 min. Next, fixed cells were permeabilized with PBS + 0.2% triton-x100 (PBS-T) and the nonspecific sites were blocked with PBS-T + normal goat serum (NGS) 10%. Cells were firstly incubated overnight with anti-SARS/SARS-CoV-2 Coronavirus Nucleocapsid Antibody (MA1-7403, Thermo Fisher Scientific) (1:100 in PBS-T + 1.5% NGS), and then with the anti-mouse Alexa Fluor 488 secondary antibody for 1 h (A11029, Invitrogen; Thermo Fisher Scientific, dilution 1:500). Finally, a mounting medium containing 40,6-diamidino-2-phenylindole (DAPI) (Fluoroshield™ with DAPI, F6057, Sigma Aldrich, Merck KGaA, Darmstadt, Germany) was employed to seal the coverslips on the glass slides (Superfrost, 10143560, Thermo Fisher Scientific). Images were then acquired with the Cytation 5 Cell Imaging Multi-Mode Reader (Biotek, Winooski, VT, USA) at 60 × (by using the Z-stack mode).

The presence of an established infection was confirmed when the cytopathic effect was observed together with viral load levels above E + 12 viral copies /mL in the supernatants, as previously reported [12]. The experiments were performed in 6 replicates in 2 experimental days.

## 3. Results

Successful viral replication was determined by quantification of the supernatants for all the tested timings (1, 2, 3, 5, 15, 30, 60 min) with MOI 1, 0.1, and 0.01; for MOI 0.001, 15, 30, and 60 min of viral absorption were able to generate an established infection; finally for MOI, 0.0001 just 60 min yield an increment in the viral load. Table 1 reports the average viral load/mL of the different tested conditions.

Morphological effects induced by SARS-CoV-2 were observed in the wells in which the molecular analysis of viral RNA indicated the occurrence of viral amplification.

**Figure 1:**
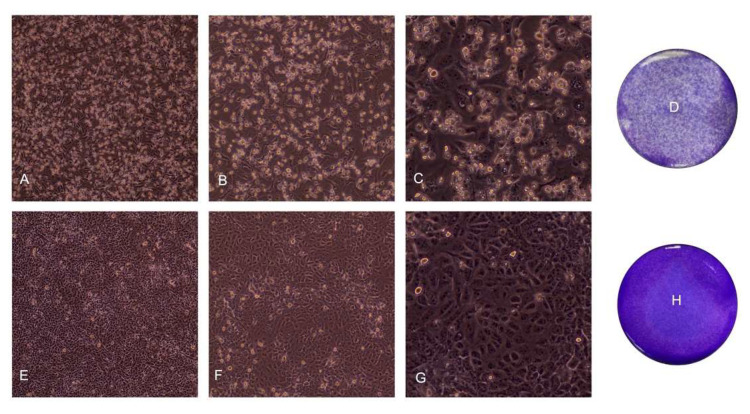
Representative images of the cytopathic effect induced by SARS-CoV-2 infection at the 7th day. Non-treated cells are shown for comparison. 10×, 20×, and 40× magnification are displayed. (**A**) Cytopathic effect induced in infected cells, 10×; (**B**) Cytopathic effect induced in infected cells, 20×; (**C**) Cytopathic effect induced in infected cells, 40× (**D**) Cytopathic effect induced in infected cells, crystal violet staining; (**E**) Non-treated cells, 10×; (**F**) Not treated cells, 20×; (**G**) Not treated cells, 40×; (**H**) Not treated cells, crystal violet staining.

Specifically, cytopathic effects, characterized by cellular rounding, detachment, and degeneration were observed (see Figure 1A–G for representative images at different magnifications). Similarly, the crystal violet staining on the 7th day revealed a significant decrease in the living cells acquiring the crystal violet dye, a sign of cell detachment from the well (representative images in Figure 1D,H).

**Figure 2:**
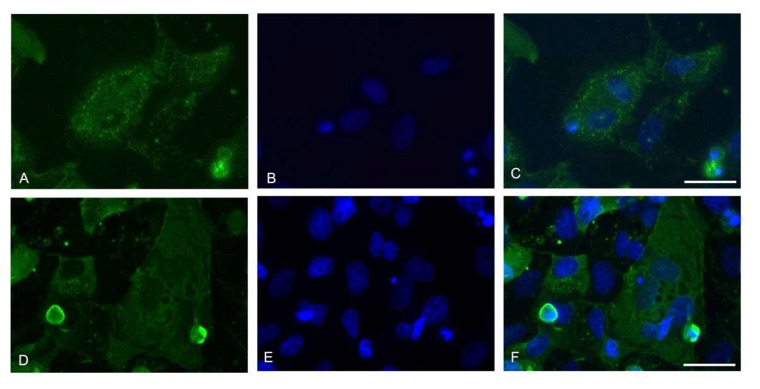
Representative images of the immunofluorescence assay for viral Nucleoprotein at the 7th day. 60× magnifications are displayed (Z-stack, scale bar 30 micron). (**A**,**D**) Nucleocapsid (green); (**B**,**E**) Nuclei (blue); (**C**,**F**) Merge of the two channels.

The nucleocapsid immunofluorescent staining showed a generalized infection of the Vero E6 monolayer with cells presenting a multinucleate phenotype (Figure 2).

## 4. Discussion

SARS-CoV-2 is still spreading around the world, reaching all the continents with a total global burden of over 207 million infected people [13,14].

Despite the worldwide mobilization of the researchers and institutions towards the study of this emerging virus, there is not a universal consensus on the viral quantity and the time of contact able to generate a persistent infection from infected individuals to their neighbors [15]. This information is important for the constitution of measures related to social distancing, contact tracing, quarantine, and medical discharge of COVID-19 affected individuals [7].

In the present work, we focused on the determination of the minimal time of absorption and the minimal quantity of virus able to produce a persistent infection in an in vitro cellular model of SARS-CoV-2 infection.

Our results highlight that in Vero E6 cell culture it is possible to establish a persistent SARS-CoV-2 infection even after a very short time of viral absorption, with only 1 min being able to generate a persistent infection with MOI 1, 0.1, and 0.01. Notably, there are no appreciable differences both in terms of viral load and cytopathic effect between infections obtained with 1, 2, 3, 5, 15, 30, or 60 min of absorption at 7 days post-infection. These results possibly suggest that the initial binding of the virus with the cells is crucial and sufficient for virions to adhere and enter the cellular membrane, with subsequent intracellular replication, including when low MOIs were tested. With lower MOI, only a longer time of viral inoculation was able to generate an infection, being 15′ and 60′ the minimal times required to yield viral replication for MOI 0.001 and 0.0001, respectively. Interestingly, the time needed to macroscopically perceive these effects is also compatible with the onset of the symptoms in COVID-19 individuals after close contact with a positive subject [16].

An evident cellular death effect was observed at the 7th day post-infection by crystal violet staining, which showed a noticeable decrease in the number of well-adhering cells. The cells were characterized by cellular rounding, detachment, and degeneration, together with the presence of multinucleate cells. This phenotype is characteristic of infected cells. Indeed, the sole presence of the viral spike protein in correspondence with the plasma membrane can lead to the formation of receptor-dependent syncytia. Nevertheless, it has been reported that Vero cells seem to form syncytia only when co-cultured with other cell lines (U2OS-ACE2 and 293T-ACE2 cell lines) and came in contact with U2OS-ACE2-infected cells or 293T-ACE2 cells expressing the spike protein. Vero cells fused also when they were transfected with the S protein, but they did not fuse upon infection. Therefore, the presence of ACE2 or S protein is essential to generate syncytia [17]. Our immunofluorescence images showed multinucleate cells upon infection; however, we did not employ a GFP–Split complementation system as in the paper by Buchrieser et al. [17]; therefore, the syncytia formation cannot be confirmed. Moreover, the Vero cell lines used in our experiments might be slightly different between the two experimental settings, since in the present study the Vero E6 clone (ATCC CRL-1586) was employed, while in the paper redacted by Buchrieser et al. [17], the ATCC code was not reported, thus not allowing a comparison. Nevertheless, the formation of multinucleate cells in Vero E6 cells simply infected with SARS-CoV-2 was previously reported [10], as in the present study.

Besides Vero cells, SARS-CoV-2 infected in vitro human airway epithelium (HAE), in line with the respiratory symptoms induced in vivo. Fully differentiated HAE are highly susceptible to viral replication, specifically the ciliated and secretory cells, with long virus persistence for 3–6 days (peak at 48–72 h). SARS-CoV-2 induced the generation of large syncytial cells, the destruction of cellular tight junction, cytopathic effect, and cellular apoptosis. It has been shown that SARS-CoV-2 infects and replicates more efficiently in primary cell lines with respect to the immortalized ones, therefore confirming its high infectious potential and transmissibility [18].

Indeed, our results, albeit conducted in an in vitro model of infection (i.e., Vero E6 cell line) that obviously does not recapitulate either the naso-oropharyngeal environment nor the lung, further corroborate the previous data regarding the high infective capacity of SARS-CoV-2, even during short-time exposures and with low quantities of viral particles. To date, the international WHO guidelines indicate 15 min as the time necessary to define close contacts with a COVID-19-affected individual [7]. The current work is aimed at analyzing and further characterizing in vitro this condition (i.e., 15 min and less of exposure). Bearing in mind the limitations of an in vitro experimental setting in trying to represent the real scenario of a virus–host contact, based on our findings, it could be reinforced that in some high-risk contexts, such as restricted closed environments with poor air circulation (e.g., elevators, crowded means of transports), the precautions to take in the presence of a COVID-19-positive individual should be maximal. Actually, the main way that SARS-CoV-2 is transmitted is from person to person through direct, indirect, or close contact with infective secretions, such as saliva and respiratory droplets [2]. The virus is also found in very low quantities in urine, stool, and blood; thus, the viral transmission through these routes is still under discussion by the scientific community, as well as the airborne spread of SARS-COV-2, which has been hypothesized but still needs to be confirmed [15].

Our in vitro findings are in line with the epidemiological studies regarding COVID-19 outbreaks reporting clusters of infection from the first individual, even when asymptomatic, following close encounters in indoor settings, including shopping malls, restaurants, public transport (bus, train, flight, ships), healthcare facilities, hospitals, elderly care settings [19,20,21], and occupational settings [22]. Another important location that needs to be strictly considered is given by scholastic environments, including infant centers, primary and secondary schools, the latter presenting higher rates of risk [23]; public transport such as buses, trains, aircraft, and taxies should be considered as well [24].

In conclusion, our in vitro study strongly indicates that the time of absorption is relevant to establishing an infection. Indeed, in the majority of the studies regarding SARS-CoV-2 infection, although having different aims from the ones investigated in our work, Vero E6 cells (and other cell lines) are exposed to the virions for 1 h [10]. However, we have observed that a minor time of viral absorption in vitro can be employed to obtain the same consistent cytopathic effect. Therefore, taken together, our findings will initially contribute to redesigning the anti-viral infection protocols, and secondly, even if obtained in a cellular model of infection, our findings will be a warning about the transmission risk even with a very short time of exposure.

## Figures and Tables

**Table 1:** The viral load at the 7th day post-infection at the different MOI (multiplicity of infection, 1, 0.1, 0.01, 0.001, 0.0001) and timing tested (1, 2, 3, 5, 15, 30, 60 min). The average viral load for mL of 6 replicates is reported. In red are depicted the conditions where an established infection was determined.

	Time	1′	2′	3′	5′	15′	30′	60′
MOI								
**MOI 1**		6.4 × 10^12^	5.1 × 10^12^	4.0 × 10^12^	7.4 × 10^12^	5.5 × 10^12^	4.5 × 10^12^	4.5 × 10^12^
**MOI 0.1**		9.2 × 10^12^	8.3 × 10^12^	7.4 × 10^12^	1.2 × 10^12^	8.2 × 10^12^	7.1 × 10^12^	7.0 × 10^12^
**MOI 0.01**		9.4 × 10^12^	7.9 × 10^12^	7.5 × 10^12^	9.0 × 10^12^	7.4 × 10^12^	8.0 × 10^12^	1.0 × 10^12^
**MOI 0.001**		1.5 × 10^9^	1.8 × 10^9^	2.0 × 10^9^	1.3 × 10^9^	7.4 × 10^12^	7.7 × 10^12^	8.2 × 10^12^
**MOI 0.0001**		2.7 × 10^6^	3.2 × 10^6^	3.7 × 10^6^	9.6 × 10^6^	3.0 × 10^6^	1.1 × 10^6^7	4.0 × 10^12^

## Data Availability

All the data used to support the findings in this study are included in the article.

## References

[1] Patel K.P., Vunnam S.R., Patel P.A., Krill K.L., Korbitz P.M., Gallagher J.P., Suh J.E., Vunnam R.R. (2020). Transmission of SARS-CoV-2: An update of current literature. Eur. J. Clin. Microbiol. Infect. Dis..

[2] Kumar M., Taki K., Gahlot R., Sharma A., Dhangar K. (2020). A Chronicle of SARS-CoV-2: Part-I—Epidemiology, diagnosis, prognosis, transmission and treatment. Sci. Total Environ..

[3] Hoffmann M., Kleine-Weber H., Schroeder S., Krüger N., Herrler T., Erichsen S., Schiergens T.S., Herrler G., Wu N.H., Nitsche A. (2020). SARS-CoV-2 Cell Entry Depends on ACE2 and TMPRSS2 and Is Blocked by a Clinically Proven Protease Inhibitor. Cell.

[4] Hou Y.J., Okuda K., Edwards C.E., Martinez D.R., Asakura T., Dinnon K.H., Kato T., Lee R.E., Yount B.L., Mascenik T.M. (2020). SARS-CoV-2 reverse genetics reveals a variable infection gradient in the respiratory tract. Cell.

[5] Hussein M., Toraih E., Elshazli R., Fawzy M., Houghton A., Tatum D., Killackey M., Kandil E., Duchesne J. (2020). Meta-analysis on serial intervals and reproductive rates for SARS-CoV-2. Ann. Surg..

[6] Bulfone T.C., Malekinejad M., Rutherford G.W., Razani N. (2020). Outdoor transmission of SARS-CoV-2 and other respiratory viruses, a systematic review. J. Infect. Dis..

[7] World Health Organization Contact Tracing in the Context of COVID-19. https://apps.who.int/iris/bitstream/handle/10665/332049/WHO-2019-nCoV-Contact_Tracing-2020.1-eng.pdf?sequence=1&isAllowed=y.

[8] Wee L.E., Conceicao E.P., Sim X.Y.J., Aung M.K., Tan K.Y., Wong H.M., Wijaya L., Tan B.H., Ling M.L., Venkatachalam I. (2020). Minimizing intra-hospital transmission of COVID-19: The role of social distancing. J. Hosp. Infect..

[9] Wang L., Fan X., Bonenfant G., Cui D., Hossain J., Jiang N., Larson G., Currier M., Liddell J., Wilson M. (2021). Susceptibility to SARS-CoV-2 of cell lines and substrates commonly used to diagnose and isolate influenza and other viruses. Emerg. Infect. Dis..

[10] Chu H., Chan J.F.-W., Yuen T.T.-T., Shuai H., Yuan S., Wang Y., Hu B., Yip C.C.-Y., Tsang J.O.-L., Huang X. (2020). Comparative tropism, replication kinetics, and cell damage profiling of SARS-CoV-2 and SARS-CoV with implications for clinical manifestations, transmissibility, and laboratory studies of COVID-19: An observational study. Lancet Microbe.

[11] Heinen N., Klöhn M., Steinmann E., Pfaender S. (2021). In Vitro lung models and their application to study SARS-CoV-2 pathogenesis and disease. Viruses.

[12] Barbieri P., Zupin L., Licen S., Torboli V., Semeraro S., Cozzutto S., Palmisani J., Di Gilio A., de Gennaro G., Fontana F. (2021). Molecular detection of SARS-CoV-2 from indoor air samples in environmental monitoring needs adequate temporal coverage and infectivity assessment. Environ. Res..

[13] Dong E., Du H., Gardner L. (2020). An interactive web-based dashboard to track COVID-19 in real time. Lancet Infect. Dis..

[14] WHO, World Health Organization WHO Coronavirus (COVID-19) Dashboard. https://covid19.who.int.

[15] World Health Organization Transmission of SARS-CoV-2: Implications for Infection Prevention Precautions. https://www.who.int/publications/i/item/modes-of-transmission-of-virus-causing-covid-19-implications-for-ipc-precaution-recommendations.

[16] Bar-On Y.M., Flamholz A., Phillips R., Milo R. (2020). SARS-CoV-2 (COVID-19) by the numbers. eLife.

[17] Buchrieser J., Dufloo J., Hubert M., Monel B., Planas D., Rajah M.M., Planchais C., Porrot F., Guivel-Benhassine F., Van der Werf S. (2020). Syncytia Formation by SARS-CoV-2-infected Cells. EMBO J..

[18] Zhu N., Wang W., Liu Z., Liang C., Wang W., Ye F., Huang B., Zhao L., Wang H., Zhou W. (2020). Morphogenesis and cytopathic effect of SARS-CoV-2 infection in human airway epithelial cells. Nat. Commun..

[19] Zhang X.S., Duchaine C. (2020). SARS-CoV-2 and health care worker protection in low-risk settings: A review of modes of transmission and a novel airborne model involving inhalable particles. Clin. Microbiol. Rev..

[20] Comber L., O Murchu E., Drummond L., Carty P.G., Walsh K.A., De Gascun C.F., Connolly M.A., Smith S.M., O’Neill M., Ryan M. (2020). Airborne transmission of SARS-CoV-2 via aerosols. Rev. Med. Virol..

[21] Leclerc Q.J., Fuller N.M., Knight L.E., Funk S., Knight G.M., CMMID COVID-19 Working Group (2020). What settings have been linked to SARS-CoV-2 transmission clusters?. Wellcome Open Res..

[22] Marinaccio A., Boccuni F., Rondinone B.M., Brusco A., D’Amario S., Iavicoli S. (2020). Occupational factors in the COVID-19 pandemic in Italy: Compensation claims applications support establishing an occupational surveillance system. Occup. Environ. Med..

[23] European Centre for Disease Prevention and Control COVID-19 in Children and the Role of School Settings in Transmission—First Update; 2020. https://www.ecdc.europa.eu/sites/default/files/documents/COVID-19-in-children-and-the-role-of-school-settings-in-transmission-first-update_1.pdf.

[24] Shen J., Duan H., Zhang B., Wang J., Ji J.S., Wang J., Pan L., Wang X., Zhao K., Ying B. (2020). Prevention and control of COVID-19 in public transportation: Experience from China. Environ. Pollut..

